# Supplementary material for: A network property necessary for concentration robustness
Source: Nat Commun. 2016 Oct 19;7:13255. doi: 10.1038/ncomms13255 (PMC5075777; doi:10.1038/ncomms13255)
Supplement: Supplementary Information — Supplementary Figures 1-7, Supplementary Tables 1-3, Supplementary Methods, Supplementary Discussion, Supplementary References. [file ncomms13255-s1.pdf]

Supplementary figure

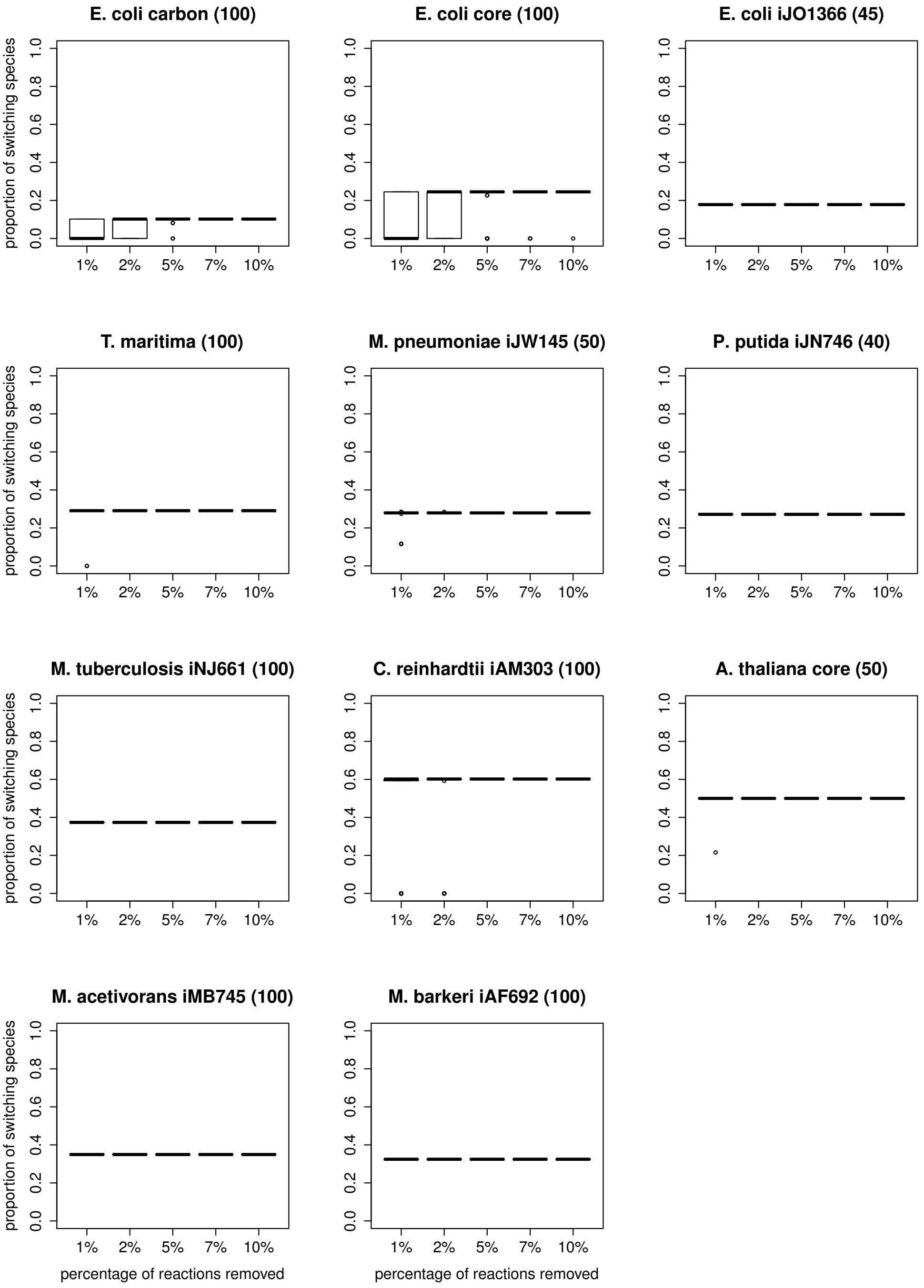

Supplementary Figure 1: **Effects of network alterations on the findings from applications of the derived necessary condition.** To investigate the effect of bias in the network, 1, 2, 5, 7 and 10% randomly selected reactions are removed from each of the analyzed networks. The effect of the reaction removal is quantified by the proportion of switching metabolites. The switching ratio for every network and perturbation level was determined over at least 40 samples (indicated next to the name of the networks analyzed).

Supplementary tables

network	domain	kingdom	number of metabolites	number of reactions*	description	reference
M acetivorans iMB745	Archea	Euryarchaeota	715	825	GSMN of *M. acetivorans*	[10]
M barkeri iAF692	Archea	Euryarchaeota	628	689	GSMN of *M. barkeri*	[6]
E coli Carbon	Bacteria	Proteobacteria	48	98	Carbon metabolism of *E. coli*	[16]
E coli Core	Bacteria	Proteobacteria	52	75	Central metabolism of *E. coli*	[14]
E coli iJO1366	Bacteria	Proteobacteria	1805	2583	GSMN of *E. coli*	[13]
M pneumoniae iJW145	Bacteria	Proteobacteria	251	214	GSMN of *M. pneumoniae*	[18]
M tuberculosis iNJ661	Bacteria		826	1025 (no info for mining)	GSMN of *M. tuberculosis*	[8]
T maritima	Bacteria		491	555	Core metabolism of *T. maritima*	[19]
P putida iJN746	Bacteria		909	1056	GSMN of *P. putida* KT2440	[12]
A niger	Eukaryota	Fungi	1177	1396	GSMN of *A. niger*	[2]
C reinhardtii AM303		Plantae	220	268	Central metabolism of *C. reinhardtii*	[11]
Arabidopsis core		Plantae	407	544	Core metabolism of *A. thaliana*	[3]
M musculus		Animalia	2110	2035 (no info for mining)	GSMN of *M. musculus*	[15]
H sapiens		Animalia	2766	2042	GSMN of *H. sapiens*	[4]

Supplementary Table 1: **Metabolite networks to which the necessary condition for ACR is applied.** The abbreviation GSMN stands for genome-scale metabolic network. The table includes the domain and kingdom of each network, the number of metabolites, reactions, deficiency, and the original source for the network. * The total number of reactions, including the blocked, is reported as obtained in the originally published network models.

network	NADH	NAD	NADP	NADPH	ATP	ADP	AMP	number of metabolites satisfying necessary condition for ACR	number of metabolites	number of reactions*	deficiency
M acetivorans iMB745								250	715	892	138
M barkeri iAF692								204	628	741	92
E coli Carbon								5	48	104	27
E coli Core	c	c	c		c			14	52	114	26
E coli iJO1366								322	1805	3243	768
M pneumoniae iJW145								85	214	321	58
M tuberculosis iNJ661								309	826	1085	209
T maritima								143	491	627	104
P putida iJN746								247	909	1314	172
A niger								765	1177	1505	309
C reinhardtii AM303			c,h		h,m,c			133	220	294	58
Arabidopsis core	c,h	c,h,p,m	m	cm,c				292	407	624	110
M musculus								1719	2110	1144	291
H sapiens		m	c,m	c,r				1735	2766	2993	794

Supplementary Table 2: **Metabolic networks for which ACR is tested**. (h=chloroplast; c=cytosol; m=mitochondria; p=peroxisome; f=flagellum). The table includes the compartments of the energy related metabolites, the number of metabolites which do not violate the necessary condition, the number of metabolites, reactions, and the deficiency of the network. * Blocked reactions are removed from the model as they preclude the existence of positive steady state. The number of reactions refer to that in the models modiefied in such way and in which reversible reactions were split into two irreversible reactions.

**Number of components satisfying necessary condition**	**Number of components satisfying necessary condition and constant concentration from simulations**	**Components satisfying necessary condition and showing invariant steady-state concentration in simulations**
784 (94.46%)	2 (0.24%)	fum_e<br>Ex_fum(e)_fum_e_complex

Supplementary Table 3: **Number of components that are 'likely' ACR.** We use the kinetic model of *E. coli* from Khodayari *et al.* [9] to determine components that are likely ACR. To this end, we find the components that show invariant steady-state concentrations over 150 perturbations of the initial concentrations. In addition, we also determine the components satisfying the necessary condition. The names of the components in the third column are as they appear in the model.

Supplementary methods

The objective of this supporting information is to provide a proof of the theorem from the main text together with illustrative applications of the theorem to some mass action networks. This piece of work is divided into four sections (including this section). The toy example in Fig. 2 will be used throughout this section to illustrate the definitions and concepts of chemical reaction networks theory useful in establishing the proof of the theorem.

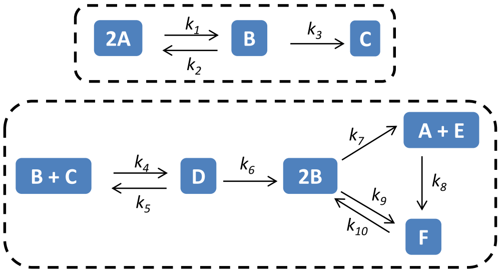

Supplementary Figure 2: **Network of biochemical reactions**. Six species, denoted with capital letters (A to F) are inter-converted through 10 irreversible reactions of rate constants (k_1 to k_{10}). Each connected component, surrounded by dashed lines, corresponds to a linkage class.

Some notations and definitions

Definition 1. *[5] A **chemical reaction network** is a triplet $\{\mathscr{S},\mathscr{C},\mathscr{R}\}$ where*

1. *$\mathscr{S}$ is a finite set of **species**,*
2. *$\mathscr{C}$ is a finite set of distinct vectors in $y \in \mathbb{R}_+^{|\mathscr{S}|}$ such that $\bigcup_{y\in\mathscr{C}} \operatorname{supp} y = \mathscr{S}$, where*

$$\operatorname{supp} y := \{j \in \mathscr{S} \,|\, y_j \neq 0\}$$

and y_j is the multiplicity of species j in complex y.

*Elements of $\mathscr{C}$ are called the **complexes** of the network.*

3. *$\mathscr{R} \subset \mathscr{C} \times \mathscr{C}$ such that :*
   (a) *for any complex $y \in \mathscr{C}$, $y \rightharpoonup y \notin \mathscr{R}$. Where $(y, y') \in \mathscr{R}$ is denoted by $y \rightharpoonup y^{'} \in \mathscr{R}$, we say that the complex y does not "**react to**" itself,*
   (b) *for each complex $y \in \mathscr{C}$, there exists a complex $y^{'} \neq y$ such that $y \rightharpoonup y^{'} \in \mathscr{R}$ or $y^{'} \rightharpoonup y \in \mathscr{R}$.*

Example : For the network in Fig. 2, the set of species is

$$\mathscr{S} = \{A, B, C, D, E, F\},$$

the set of complexes is

$$\mathscr{C} = \{\{2A\}, \{B\}, \{C\}, \{B + C\}, \{D\}, \{2B\}, \{A + E\}, \{F\}\}$$

and the set of reactions is

$$\mathscr{R} = \{\{2A \rightharpoonup B\}, \{B \rightharpoonup 2A\}, \{B \rightharpoonup C\}, \{B + C \rightharpoonup D\}, \{D \rightharpoonup B + C\},$$
$$\{D \rightharpoonup 2B\}, \{2B \rightharpoonup A + E\}, \{A + E \rightharpoonup F\}, \{2B \rightharpoonup F\}, \{F \rightharpoonup 2B\}\}.$$

Remark 2. *Note that the condition imposed in Definition 1 3.(a) asserts that the cases such as $y \rightharpoonup y$ should be removed before consideration of a chemical reaction network, since they do not affect the dynamic of the network.*

Definition 3. *[5] Let $\{\mathscr{S}, \mathscr{C}, \mathscr{R}\}$ be a chemical reaction network. A **kinetics** k is an assignment to each reaction $y \to y^{'} \in \mathscr{R}$ of a **rate function** $V_{y\to y'}(\cdot)$. In other words, k is the function defined as follows:*

$$\begin{aligned} k: \quad \mathscr{R} &\longrightarrow \mathbb{R}_+^{\mathbb{R}_+^{\mathscr{S}}} \\ y \to y^{'} &\longmapsto V_{y\to y'}(\cdot), \end{aligned} \tag{1}$$

where $\mathbb{R}_+^{\mathbb{R}_+^{\mathscr{S}}}$ is the set of functions from $\mathbb{R}_+^{\mathscr{S}}$ to $\mathbb{R}_+$.

Definition 4. *[5] A **reaction system** $\{\mathscr{S}, \mathscr{C}, \mathscr{R}, k\}$ is a chemical reaction network $\{\mathscr{S}, \mathscr{C}, \mathscr{R}\}$ endowed with a kinetics k.*

Definition 5. *[5] The kinetics k of a reaction system $\{\mathscr{S}, \mathscr{C}, \mathscr{R}, k\}$ is **mass action** if, for every $y \rightharpoonup y^{'} \in \mathscr{R}$ and $c \in \mathbb{R}_+^{\mathscr{S}}$, there exists a positive number $k_{y\rightharpoonup y'}$ such that the rate function is defined as follows:*

$$V_{y\rightharpoonup y'}(c) = k_{y\rightharpoonup y'} c_y. \tag{2}$$

*The positive real $k_{y\rightharpoonup y'}$ is called the **rate constant** of the reaction $y \rightharpoonup y^{'}$ and c_y is the concentration of the complex y defined in this case as follows:*

$$c_y = \prod_{j\in\mathscr{S}} c_j^{y_j}. \tag{3}$$

Definition 6. *Let $T = \{\mathscr{S}, \mathscr{C}, \mathscr{R}, k\}$ be a reaction system and $S \in \mathscr{S}$. We will refer to $T_{-S} = \{\mathscr{S}_{-S}, \mathscr{C}_{-S}, \mathscr{R}_{-S}, k_{-S}\} \neq T$ as the **reaction system obtained upon removal of species** S, which we define as the reaction system satisfying the following properties:*

1. *$\mathscr{S}_{-S}$ is a finite set of species such that $\mathscr{S}_{-S} = \mathscr{S} \backslash S$,*
2. *$\mathscr{C}_{-S}$ is a finite set of distinct vectors in $\mathbb{R}_+^{|\mathscr{S}|-1}$ whose elements are obtained by removing S from each complex containing S in T,*
3. *$\mathscr{R}_{-S} \subset \mathscr{C}_{-S} \times \mathscr{C}_{-S}$ is as in Definition 1 (3) for $\mathscr{C}_{-S} \times \mathscr{C}_{-S}$.*
4. *The kinetics k_{-S} in T_{-S} is the same as the kinetics in T with different rate constants.*

Remark 7. *1. If T is a closed system, T_{-S} may be closed or open. The rate constants of T_{-S} can be determined (see below).*

2. The number of reactions in T_{-S} is different from the number of reactions in T if S is involved in a reaction whose difference between the product and substrate complexes is a non-zero multiple of S (e.g. $y \rightharpoonup y + S$). In this case, the removal of S from T will lead to reactions in which the product and substrate complexes are the same ($y \rightharpoonup y$), which should be removed in order the deal with a well defined chemical reaction network (see Remark 2).

Note that, the number of reactions in T_{-S} is as well different from the number of reactions in T if the removal of species S from T yields the occurrence of duplicate reactions. However, in this case the dynamic of the system is affected since each reaction is endowed with a rate constant (e.g. reactions $y \xrightharpoonup{k} y'$ and $y \xrightharpoonup{k'} y'$.).

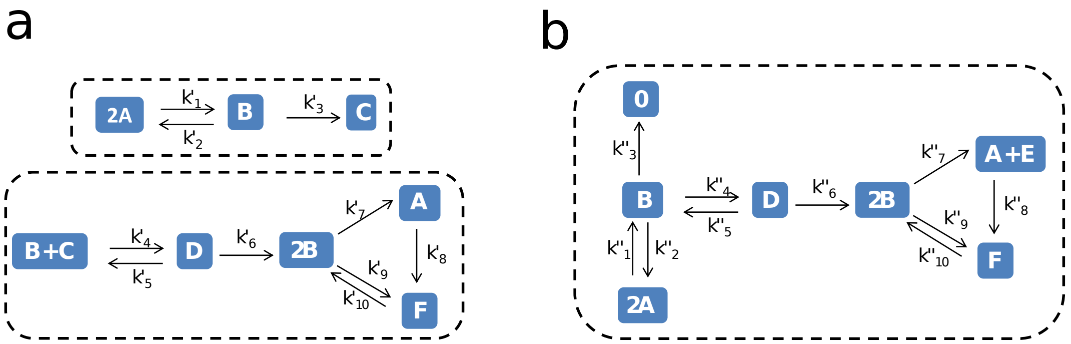

Supplementary Figure 3: **Illustration of the networks obtained upon removal of a species.** **(a)** Network of biochemical reactions with rate constants k_1' to k_{10}', obtained upon removal of species E from the biochemical reaction network in Fig. 2. **(b)** Network of biochemical reactions with rate constants k_1'' to k_{10}'', obtained upon removal of species C from the biochemical reaction network in Fig. 2. Removal of species C yields the inclusion of the zero complex denoted by 0.

Definition 8. *[5] Two complexes $y, y^{'} \in \mathscr{C}$ are* ***directly linked*** *(or $y \longleftrightarrow y^{'}$) if $y \rightharpoonup y^{'}$ or $y^{'} \rightharpoonup y$. These complexes are* ***linked*** *(or $y \sim y^{'}$) if any of the following conditions are satisfied*

1. $y = y^{'}$,
2. $y \longleftrightarrow y^{'}$,
3. $\mathscr{C}$ *contains a sequence* $\{y_1, y_2, \cdots, y_k\}$ *such that,*

$$y \longleftrightarrow y_1 \longleftrightarrow y_3 \cdots y_k \longleftrightarrow y^{'}.$$

The equivalence relation $\sim$ induces a partition of $\mathscr{C}$ into a family of equivalence classes $\{L_\theta\}$, called ***linkage classes****.*

Example: For the network in Fig. 2, the set of linkage classes is

$$L : \{\{2A, B, C\}, \{B + C, D, 2B, A + E, F\}\}.$$

Definition 9. *[5] A complex $y \in \mathscr{C}$* ***ultimately reacts to*** *$y^{'} \in \mathscr{C}$ (or $y \Longrightarrow y^{'}$) if any of the following conditions are satisfied:*

1. $y = y^{'}$,
2. $y \rightharpoonup y^{'}$,
3. $\mathscr{C}$ *contains a sequence* $\{y_1, y_2, \cdots, y_k\}$ *such that,*

$$y \rightharpoonup y_1 \rightharpoonup y_3 \cdots y_k \rightharpoonup y^{'}.$$

The complexes y and $y^{'}$ are ***strongly linked*** *(or $y \approx y^{'}$) if $y \Longrightarrow y^{'}$ and $y^{'} \Longrightarrow y$.*

The equivalence relation $\approx$ induces a partition of $\mathscr{C}$ into a family of equivalence classes $\{\Lambda_\varrho\}$, called ***strong linkage classes****.*

The set of strong linkage classes in Fig. 2 is

$$\Lambda : \{\{2A, B\}, \{C\}, \{B + C, D\}, \{2B, A + E, F\}\}.$$

Definition 10. *[5] A reaction system is* ***weakly reversible*** *if for the given network, the relation ultimately reacts to ($\Longrightarrow$) is symmetric, i.e., if $y \Longrightarrow y^{'}$ then $y^{'} \Longrightarrow y$.*

Definition 11. *[5] A* ***terminal strong linkage class*** *is a strong linkage class Λ with property that no complex in Λ reacts to a complex in a different strong linkage class.*

The set of terminal strong linkage classes

$$T\Lambda : \{\{C\}, \{2B, A + E, F\}\}.$$

Definition 12. *The **reaction vector** of a chemical reaction in $T = \{\mathscr{S}, \mathscr{C}, \mathscr{R}, k\}$ is the vector difference between the right and left complex of the given reaction. In other words, reaction vectors are elements of the following set*

$$\{y^{'} - y \in \mathbb{R}^{|\mathscr{C}|} \,|\, y \rightharpoonup y^{'} \in \mathscr{R}\}.$$

*The reaction vectors are the columns of the **stoichiometric matrix** N of the reaction system T. The rank of the reaction system $\{\mathscr{S}, \mathscr{C}, \mathscr{R}, k\}$, denoted by r, is the rank of its set of reaction vectors. The span of the reaction vectors is called the **stoichiometric subspace**, which we denote $\mathscr{V}$.*

One of the central concept of this work is that of deficiency, defined as follows:

Definition 13. *[5] Let n, l, r be the number of complexes, the number of linkage classes and the rank of a reaction system $T = \{\mathscr{S}, \mathscr{C}, \mathscr{R}, k\}$, respectively. The **structural deficiency** of T, is a non-negative integer δ_s defined as follows:*

$$\delta_s = n - l - r \tag{4}$$

*and the **dynamic deficiency** δ_d of T is given by*

$$\delta_d = \dim[\ker(Y) \cap \mathrm{Im}(A_k)], \tag{5}$$

where Y is a linear map and A_k is a non-linear map defined as follows [7]:

$$\begin{aligned} Y : \mathbb{R}^{|\mathscr{C}|} &\longrightarrow \mathbb{R}^{|\mathscr{S}|} \\ w_y &\longmapsto Y(w_y) = y \end{aligned} \tag{6}$$

and

$$\begin{aligned} A_k : \mathbb{R}^{|\mathscr{C}|} &\longrightarrow \mathbb{R}^{|\mathscr{C}|} \\ x &\longmapsto A_k(x) = \sum_{y \to y' \in \mathscr{R}} k_{y \to y'} x_y (w_{y'} - w_y), \end{aligned} \tag{7}$$

where $k_{y \rightharpoonup y'}$ is the rate constant of any reaction $y \rightharpoonup y^{'} \in \mathscr{R}$, $\{w_y, y \in \mathscr{C}\}$ represent the set of vectors of the natural basis of $\mathbb{R}^{|\mathscr{C}|}$ and $x_y, y \in \mathscr{C}$ are the components of $x \in \mathbb{R}^{|\mathscr{C}|}$.

Proposition 14. *For a reaction system $T = \{\mathscr{S}, \mathscr{C}, \mathscr{R}, k\}$,*

$$\delta_s = \dim \ker(Y \restriction_{\mathscr{Q}}), \tag{8}$$

where $\mathscr{Q} = span\{w_{y'} - w_y \,|\, y \rightharpoonup y^{'}\}$ and $Y \restriction_{\mathscr{Q}}$ is the function Y restricted to $\mathscr{Q}$, with Y defined as in Eq. (6).

Proof. It can be shown that the function Y is a surjection from $\mathscr{Q}$ to the stoichiometric subspace $\mathscr{V}$. By applying the rank-nullity theorem on the function $Y \restriction_{\mathscr{Q}}$, we have that

$$\begin{aligned} \dim \ker(Y \restriction_{\mathscr{Q}}) &= \dim \mathscr{Q} - \dim \mathrm{Im}(Y \restriction_{\mathscr{Q}}) \\ &= \dim \mathscr{Q} - r, \end{aligned} \tag{9}$$

where $r = rank(\mathscr{V})$. From [1] (an alternative proof has been provided by Gunawardena in [7]) the dimension of $\mathscr{Q}$ corresponds to the rank of the incidence matrix A of the system, which is the difference between the number of vertices (complexes) and the number of connected components (linkage classes) of the network. This implies,

$$\dim \mathscr{Q} = n - l, \tag{10}$$

where n and l are the number of complexes and linkage classes, respectively. Thus, substituting Eq. (10) in (9) yields $\dim \ker(Y \restriction_{\mathscr{Q}}) = n - l - r = \delta_s$. □

Remark 15. *The dynamic deficiency of a reaction system $T = \{\mathscr{S}, \mathscr{C}, \mathscr{R}, k\}$ is smaller than or equal to the structural deficiency of T, i.e.,*

$$\delta_d \leq \delta_s. \tag{11}$$

Proof. Since $\mathrm{Im}(A_k) \subset \mathscr{Q}$, we have that

$$\ker Y \cap \mathrm{Im}(A_k) \subset \ker Y \cap \mathscr{Q}, \Longrightarrow \ker Y \cap \mathrm{Im}(A_k) \subset \ker(Y \restriction_{\mathscr{Q}}), \tag{12}$$

thus,

$$\delta_d = \dim[\ker Y \cap \mathrm{Im}(A_k)] \leq \dim[\ker(Y \restriction_{\mathscr{Q}})] = \delta_s. \tag{13}$$

□

Moreover, the dynamic deficiency and the structural deficiency coincide in a reaction system if and only if the number of terminal strong linkage classes is equal to the number of linkage classes of the system (see [7] for a proof).

Definition 16. *[5] The* ***species-formation rate function*** *of a reaction operating under a mass action kinetic $T = \{\mathscr{S}, \mathscr{C}, \mathscr{R}, k\}$, is the function*

$$\begin{aligned} f(.,k) : \mathbb{R}^{|\mathscr{S}|} &\longrightarrow \mathbb{R}^{|\mathscr{S}|} \\ c &\longmapsto f(c,k) = \sum_{y \to y' \in \mathscr{R}} k_{y \to y'} c_y (y' - y), \end{aligned} \tag{14}$$

where $k_{y \to y'}$ corresponds to the rate constant associated to reaction $y \rightharpoonup y'$ and $c_y = \prod_{j \in \mathscr{S}} {c_j}^{y_j}$ is a convenient way to represent mass action.

A ***steady state concentration vector*** *of the reaction system $T = \{\mathscr{S}, \mathscr{C}, \mathscr{R}, k\}$ is a non-negative element $c \in \mathbb{R}^{|\mathscr{S}|}$ such that*

$$f(c,k) = 0 \tag{15}$$

and a ***positive steady state concentration vector*** *is a positive element $c \in \mathbb{R}_+^{*|\mathscr{S}|}$ that satisfies Eq.* (15)*.*

In the next lines, instead of positive steady state concentration we will use the shorter positive steady state.

Definition 17. *[5] The system of ordinary differential equations describing the rate of change of species concentrations c in a mass action system $T = \{\mathscr{S}, \mathscr{C}, \mathscr{R}, k\}$ is given by*

$$\frac{dc}{dt} = f(c, k), \tag{16}$$

where f is the species-formation rate function.

Another central concept of this work is that of absolute concentration robustness, defined as follows:

Definition 18. *[17] A reaction system $\{\mathscr{S}, \mathscr{C}, \mathscr{R}, k\}$ has **absolute concentration robustness (ACR)** in a species $S \in \mathscr{S}$ if the system has a positive steady state c^*, and if for every other positive equilibrium $c^{**}, c^{**}_S = c^*_S$.*

Proof of the theorem

The principal result in the main text provides a necessary (see Theorem 25) condition that an ACR species must satisfy. The proof of the theorem will require two main steps, described as follows:

1. We will first prove that removal of a species S with ACR from a system $T = \{\mathscr{S}, \mathscr{C}, \mathscr{R}, k\}$ does not affect the dynamic deficiency of T (see Proposition 19).
2. Then, since the dynamic deficiency depends on the rate constant at which the system has a positive steady state, its calculation is a non-trivial task as often not all rate constants are known. To overcome this issue, we show that the property proven for the dynamic deficiency also holds for the structural deficiency.

   To arrive at these results, we show an isomorphism between the vector spaces whose dimension is the dynamic deficiency, with and without removal of a species (see Proposition 19), as well as the isomorphism between the vector spaces whose dimension is the structural deficiency, with and without removal of the species (see Proposition 23).

Without loss of generality, we assume that the set of species $\mathscr{S}$

is ordered according to the relation "$\curvearrowright$" which is defined as follows:

$$\forall S_1, S_2 \in \mathscr{S} : S_1 \curvearrowright S_2 \iff j_{S_1} \leq j_{S_2},$$

where j_{S_1} and j_{S_2} are the indexes of the species S_1 and S_2 in the set of species, $\mathscr{S}$.

Throughout this work, we assume that our mass action reaction system has a positive steady state. The proof of our theorem requires the proof of some propositions and lemmas.

Proposition 19. *Let $T = \{\mathscr{S}, \mathscr{C}, \mathscr{R}, k\}$ be a reaction system in which there exists a species S that shows ACR. The reaction system T_{-S} obtained upon removal of species S from T has the same dynamic deficiency as T.*

The proof of Proposition 19 requires the introduction of additional mappings and Lemma 20.

Let us consider $T = \{\mathscr{S}, \mathscr{C}, \mathscr{R}, k\}$ and $T_{_{-S}} = \{\mathscr{S}_{_{-S}}, \mathscr{C}_{_{-S}}, \mathscr{R}_{_{-S}}, k_{-S}\}$ the reaction systems with and without species S, respectively.

Let ψ be the function that maps species to the vectors complexes defined as follows:

$$\begin{aligned} \psi : \mathbb{R}^{|\mathscr{S}|} &\longrightarrow \mathbb{R}^{|\mathscr{C}|} \\ c &\longmapsto \psi(c) = \sum_{y \in \mathscr{C}} c_y w_y, \end{aligned} \tag{17}$$

where $c_y = \prod_{j \in \mathscr{S}} c_j{}^{y_j}$, with y_j being the multiplicity of the $j-$th species in complex y.

From the function A_k, defined as in Eq. (7), we have the following relations:

$$\begin{aligned} A_k(\psi(c)) = A_k(\sum_{y \in \mathscr{C}} c_y w_y) &= \sum_{y \to y' \in \mathscr{R}} k_{y \to y'} c_y (w_{y'} - w_y), \\ &= \sum_{y \to y' \in \mathscr{R}} k_{y \to y'} \prod_{j \in \mathscr{S}} c_j{}^{y_j} (w_{y'} - w_y), \\ &= \sum_{y \to y' \in \mathscr{R}} k_{y \to y'} c_s{}^{y_s} \prod_{j \in \mathscr{S}, j \neq s} c_j{}^{y_j} (w_{y'} - w_y), \quad S \text{ is the } s\text{−th species} \\ &= A_{k'}(\psi^{'}(c_{_{-S}})), \end{aligned}$$

where $c_{_{-S}}$ is the concentration vector obtained by removing the $s-$th component (which corresponds to the concentration of species S) from the concentration vector c. The function $\psi^{'}$ is defined as follows

$$\begin{aligned} \psi^{'} : \mathbb{R}^{|\mathscr{S}|-1} &\longrightarrow \mathbb{R}^{|\mathscr{C}|} \\ c &\longmapsto \psi^{'}(c) = \sum_{y \in \mathscr{C}} c_y w_y. \end{aligned} \tag{18}$$

Hence,

$$A_k(\psi(c)) = A_{k'}(\psi^{'}(c_{_{-S}})), \quad \text{for any species concentration vector}\, c \in \mathbb{R}^{|\mathscr{S}|}, \tag{19}$$

where $k^{'} = k_{y \rightharpoonup y'} c_S{}^{y_s}$, with $k_{y \to y'}$ being the rate constant of the reaction $y \rightharpoonup y^{'}$, c_S the concentration of the species with ACR, S, y_s the multiplicity of species S in complex y and $c_{_{-S}}$ the concentration vector obtained upon removal of the $s-$th entry from c.

Intuitively, we define

$$k_{_{-S}} = k^{'}. \tag{20}$$

Note that each $k_{y \rightharpoonup y'}$ in T maps to a unique $k_{_{-S}}$ in $T_{_{-S}}$ for every reaction $y \rightharpoonup y^{'}$.

The transformation provided by Eq. (19), only the set of species is affected; thus, the mappings given by Eqs. (6) and (7) will be modified accordingly to define the dynamic deficiency of $T_{_{-S}}$.

We define the deficiency of $T_{_{-S}}$ as $\delta_d^S = \dim[\ker(Y^S) \cap \mathrm{Im}(A_k^S)]$, where Y^S and A_k^S are given by:

$$\begin{aligned} Y^S : \mathbb{R}^{|\mathscr{C}|} &\longrightarrow \mathbb{R}^{|\mathscr{S}_S|} \\ w_z &\longmapsto Y^S(w_z) = Dz, \end{aligned} \tag{21}$$

where D is the $p-1 \times p$ matrix obtained upon removal of the S-th row of the identity matrix I_p, with $p = \dim \mathscr{S}$ and

$$\begin{aligned} A_k^S : \mathbb{R}^{|\mathscr{C}|} &\longrightarrow \mathbb{R}^{|\mathscr{C}|} \\ \theta &\longmapsto A_k^S(\theta) = \sum_{y \rightharpoonup y' \in \mathscr{R}} k_{y \rightharpoonup y'} c_S^{y_S} \theta_y (w_{y'} - w_y), \end{aligned} \tag{22}$$

where c is such that $\psi(c) = \theta$ and c_S is the concentration of species S. Note that

$$A_k^S(\theta) = A_{k_{-S}}(\theta).$$

Lemma 20. *Under the assumption of Proposition 19, the function*

$$\begin{array}{rcl} h : \ker(Y) \cap \mathrm{Im}(A_k) & \longrightarrow & \ker(Y^S) \cap \mathrm{Im}(A_k^S) \\ A_k(\psi(c)) & \longmapsto & A_k^S(\psi^{'}(Dc)), \end{array}$$

is a bijection, where ψ and $\psi^{'}$ are defined as in Eq. (17) *and Eq* (18)*, respectively. The matrix D is as in Eq.* (21)*.*

Proof. First, we need to ensure the well-definedness of h.

To prove that h is well-defined, we have to show that

$$A_k(\psi(c)) \in \ker(Y) \cap \mathrm{Im}(A_k) \Longrightarrow A_k^S(\psi^{'}(Dc)) \in \ker(Y^S) \cap \mathrm{Im}(A_k^S)$$

By definition,

$$\begin{array}{rcl} A_k(\psi(c)) \in \ker(Y) \cap \mathrm{Im}(A_k) & \Longrightarrow & Y(A_k(\psi(c))) = 0, \\ & \Longrightarrow & Y(A_k^S \psi^{'}(c_{-S})) = 0, \quad \text{from Eq. (18)}, \\ & \Longrightarrow & Y(A_k^S \psi^{'}(Dc)) = 0, \\ & \Longrightarrow & \left[Y^S(A_k^S \psi^{'}(Dc)),\, Y_{S\cdot}(A_k^S \psi^{'}(Dc) \right]^T = 0, \\ & \Longrightarrow & Y^S(A_k^S \psi^{'}(Dc)) = 0, \end{array} \tag{23}$$

where $Y_{S\cdot}$ is the row corresponding to the species S in the matrix Y.

From Eq. (23), $A_k^S \psi^{'}(Dc) \in \ker(Y^S) \cap \mathrm{Im}(A_k^S)$. Therefore h is well-defined.

Now, let us prove that h is a bijection.

Let $A_k^S \psi^{'}(c^{'})$ be an element in $\ker(Y^S) \cap \mathrm{Im}(A_k^S)$. Our aim is to show that there exists a unique $A_k \psi(c_1) \in \ker(Y) \cap \mathrm{Im}(A_k)$ such that

$$h(A_k \psi(c_1)) = A_k^S \psi^{'}(c^{'}). \tag{24}$$

From hypothesis, we know that the species S is ACR. This implies that for every positive steady state c^*, $c_S^* = \rho$, where ρ is a positive constant.

Let us set $c_1 = (c^{'}, \rho)$. We have that

$$h(A_k \psi(c_1)) = h(A_k \psi(c^{'}, \rho)) = A_k^S \psi^{'}(D(c^{'}, \rho)). \tag{25}$$

Since ρ is the entry corresponding to the species S,

$$D(c^{'}, \rho) = c^{'}. \tag{26}$$

By substituting Eq. (26) in Eq. (25), we have Eq. (24).

Our next step is to show that $A_k \psi(c_1)$ is unique. Let us assume that there exists another element $A_k \psi(c_2)$ in $\ker(Y) \cap \mathrm{Im}(A_k)$ such that

$$A_k \psi(c_1) \neq A_k \psi(c_2) \tag{27}$$

and

$$h(A_k\psi(c_1)) = h(A_k\psi(c_2)). \tag{28}$$

$$h(A_k\psi(c_1)) = h(A_k\psi(c_2)) \Longrightarrow A_k^S\psi^{'}(Dc_1) = A_k^S\psi^{'}(Dc_2), \tag{29}$$

Eq. (29) implies the following equalities:

$$\begin{aligned}
\sum_{y\to y'\in\mathscr{R}} k_{y\to y'} c_{1S}^{y_S}[Dc_1]^y(w_{y'}-w_y) &= \sum_{y\to y'\in\mathscr{R}} k_{y\to y'} c_{2S}^{y_S}[Dc_2]^y(w_{y'}-w_y),\\
\sum_{y\to y'\in\mathscr{R}} k_{y\to y'} c_{1S}^{y_S} \prod_{j\in\mathscr{S},j\neq s} {c_{1_j}}^{y_j}(w_{y'}-w_y) &= \sum_{y\to y'\in\mathscr{R}} k_{y\to y'} c_{2S}^{y_S} \prod_{j\in\mathscr{S},j\neq s} {c_{2_j}}^{y_j}(w_{y'}-w_y),\\
\sum_{y\to y'\in\mathscr{R}} k_{y\to y'} \prod_{j\in\mathscr{S}} {c_{1_j}}^{y_j}(w_{y'}-w_y) &= \sum_{y\to y'\in\mathscr{R}} k_{y\to y'} \prod_{j\in\mathscr{S}} {c_{2_j}}^{y_j}(w_{y'}-w_y),\\
A_k\psi(c_1) &= A_k\psi(c_2). \qquad (30)
\end{aligned}$$

Eq. (30) contradicts Eq.(27); hence, $A_k\psi(c_1)$ is unique and h is a bijection. □

Now, let us proceed with the proof of Proposition 19.

Proof. To prove Proposition 19 we have to show that $\delta_d = \delta_d^S$, *i.e.*, $\dim[\ker(Y)\cap \mathrm{Im}(A_k)] = \dim[\ker(Y^S)\cap \mathrm{Im}(A_k^S)]$, which we have from Lemma 20.

Therefore, if the species S shows ACR,

$$\dim[\ker(Y^S)\cap \mathrm{Im}(A_k^S)] = \dim[\ker(Y)\cap \mathrm{Im}(A_k)] \Longleftrightarrow \delta_d = \delta_d^S.$$

□

A direct consequence of Proposition 19 is the following corollary:

Corollary 21. *Let $T = \{\mathscr{S},\mathscr{C},\mathscr{R},k\}$ be weakly reversible reaction system which contains a species S with ACR. Removal of species S from T does not alter the structural deficiency δ_s of T.*

Proof. Let δ_s^S be the structural deficiency of the network obtained upon removal of S from T. According to Proposition 19, T and T_{-S} have the same dynamic deficiency. As T is weakly reversible, every strong linkage class is terminal. Moreover, removal of S keeps the network weakly reversible. Therefore, in both T and T_{-S}, the dynamic deficiency coincide with the structural deficiency (see Remark 15). Thus, $\delta_s = \delta_s^S$. □

For networks in which the terminal strong linkage classes coincide with the linkage classes, Proposition 19 remains applicable and we have the following corollary

Corollary 22. *Let $T = \{\mathscr{S}, \mathscr{C}, \mathscr{R}, k\}$ be a reaction system in which each linkage class has precisely one terminal strong linkage class and there exists a species S that shows ACR. If the reaction system T_{-S} obtained upon removal of species S from T has precisely one terminal strong linkage class per linkage class, then T and T_{-S} have the same structural deficiencies.*

Proof. Let $T = \{\mathscr{S}, \mathscr{C}, \mathscr{R}, k\}$ be a reaction system in which the terminal strong linkage classes coincide with the linkage classes and there exists a species S that shows ACR. From Proposition 19, T and T_{-S} have the same dynamic deficiencies. Since from Remark 15, the dynamic and structural deficiencies of T are equal, T and T_{-S} have the same structural deficiencies. □

However, in the case where there exists a linkage class that includes more than one terminal strong linkage class, one needs to know the values of the rate constants for which the system has a positive steady state and no restriction on the structure (e.g. the number of terminal strong linkage classes per linkage classes) of the system is required. In the following, we strengthen this result by overcoming the issue of requiring the values for the rate constants.

Proposition 23. *Let $T = \{\mathscr{S}, \mathscr{C}, \mathscr{R}, k\}$ be a reaction system containing an ACR species S. Let $T_{-S} = \{\mathscr{S}_{-S}, \mathscr{C}_{-S}, \mathscr{R}_{-S}, k_{-S}\}$ be the system obtained upon removal of species S from T. The structural deficiencies of T and T_{-S} are equal.*

Before proceeding with the proof, we will first prove the following lemma

Lemma 24. *Let $\mathscr{Q}^S$ be defined as follows:*

$$\mathscr{Q}^S = \operatorname{span}\{M(w_{y'} - w_y) \,|\, y \rightharpoonup y^{'} \in \mathscr{R}\}, \tag{31}$$

where, M is the matrix that removes the rows corresponding to the duplicated complexes from the complex-identity matrix.

Under the assumption of Proposition 23, the function

$$\begin{aligned} g : \mathscr{Q} &\longrightarrow \mathscr{Q}^S \\ \beta &\longmapsto M\beta \end{aligned}$$

is a bijection, where $\mathscr{Q}$ is as above (see Proposition 14).

Proof. By definition of $\mathscr{Q}$ and $\mathscr{Q}^S$ is well-defined. Let $x^S \in \mathscr{Q}^S$, we have to show that there exists a unique element $x \in \mathscr{Q}$ such that $g(x) = x^S$.

Since $x^S \in \mathscr{Q}^S$, there exists $({x^S}_{y \to y'}, y \to y^{'} \in \mathscr{R})$ such that

$$x^S = \sum_{y \to y' \in \mathscr{R}} {x^S}_{y \rightharpoonup y'} M(w_{y'} - w_y). \tag{32}$$

For $x = \sum_{y \to y' \in \mathscr{R}} {x^S}_{y \rightharpoonup y'} (w_{y'} - w_y)$, we have that $x \in \mathscr{Q}$ and $g(x) = x^S$.

Let us prove the uniqueness of such x. Thus, let us assume that there exists another element $z \in \mathscr{Q}$ such that $g(x) = g(z)$.

Since x and z belong to $\mathscr{Q}$, there exist $(x_{y\to y'}, y \to y' \in \mathscr{R})$ and $(z_{y\to y'}, y \to y' \in \mathscr{R})$ such that

$$x = \sum_{y\to y'\in\mathscr{R}} x_{y\to y'}(w_{y'} - w_y) \tag{33}$$

and

$$z = \sum_{y\to y'\in\mathscr{R}} z_{y\to y'}(w_{y'} - w_y). \tag{34}$$

$$\begin{aligned} g(x) = g(z) \quad &\Longrightarrow \quad \sum_{y\to y'\in\mathscr{R}} x_{y\to y'} M(w_{y'} - w_y) = \sum_{y\to y'\in\mathscr{R}} z_{y\to y'} M(w_{y'} - w_y), \\ &\Longrightarrow \quad M \sum_{y\to y'\in\mathscr{R}} x_{y\to y'}(w_{y'} - w_y) = M \sum_{y\to y'\in\mathscr{R}} z_{y\to y'}(w_{y'} - w_y). \end{aligned}$$

Since the matrix M is full row rank, its pseudo inverse M^+ exists. By multiplying on the left, the right and left hand side of Eq. (35) by M^+, we have from Eqs. (33) and (34), that $x = z$, hence the uniqueness of x. Therefore, the bijectivity of g. □

Now, we proceed with the proof of Proposition 23.

Proof. Let $T = \{\mathscr{S}, \mathscr{C}, \mathscr{R}, k\}$ be a reaction system and $T_S^* = \{\mathscr{S}_S^*, \mathscr{C}_S^*, \mathscr{R}_S^*, k_S^*\}$ the system obtained upon removal of a species S from T. In order to show that the structural deficiencies are not altered, we need to prove that $\dim\ker(Y \restriction_{\mathscr{Q}})$ and $\dim\ker(Y^S \restriction_{\mathscr{Q}^S})$ are the same.

From the rank-nullity theorem, we have the following relation

$$\dim\ker(Y^S \restriction_{\mathscr{Q}^S}) = \dim \mathscr{Q}^S - \dim \mathrm{Im}(Y^S \restriction_{\mathscr{Q}^S}). \tag{35}$$

Since, the function Y^S is a surjection from $\mathscr{Q}^S$ to the stoichiometric subspace in T_{-S}, $\mathscr{V}^S$ and, T and T_{-S} have the same number of reaction, we have that

$$\dim \mathrm{Im}(Y^S \restriction_{\mathscr{Q}^S}) = rank(\mathscr{V}^S) = rank(\mathscr{V}) = r. \tag{36}$$

From Lemma 24, we have that

$$\dim \mathscr{Q}^S = \dim \mathscr{Q}. \tag{37}$$

By substituting Eqs. (36) and (37) in Eq. (35) we obtain that

$$\begin{aligned} \dim\ker(Y^S \restriction_{\mathscr{Q}^S}) \quad &= \quad \dim \mathscr{Q} - r & (38) \\ &= \quad \delta_s. & (39) \end{aligned}$$

Therefore T and T_{-S} have the same structural deficiencies. □

We can now state our main result which is a necessary condition for the existence of an ACR species in a reaction system

Theorem 25. *Let $T = \{\mathscr{S}, \mathscr{C}, \mathscr{R}, k\}$ be a mass action reaction system which for given rate constants admits a positive steady state with and without removal of S. If the system T has absolute concentration robustness in species S, then the systems with and without removal of S have the same structural deficiencies.*

Proof. Consequence of Propositions 19 and 23. □

Supplementary discussion

This section is devoted to illustrate the theorem in the main text by providing application to some examples.

Let us consider the deficiency one network in Fig. 4c. From Fig. 4a and b we see that that the

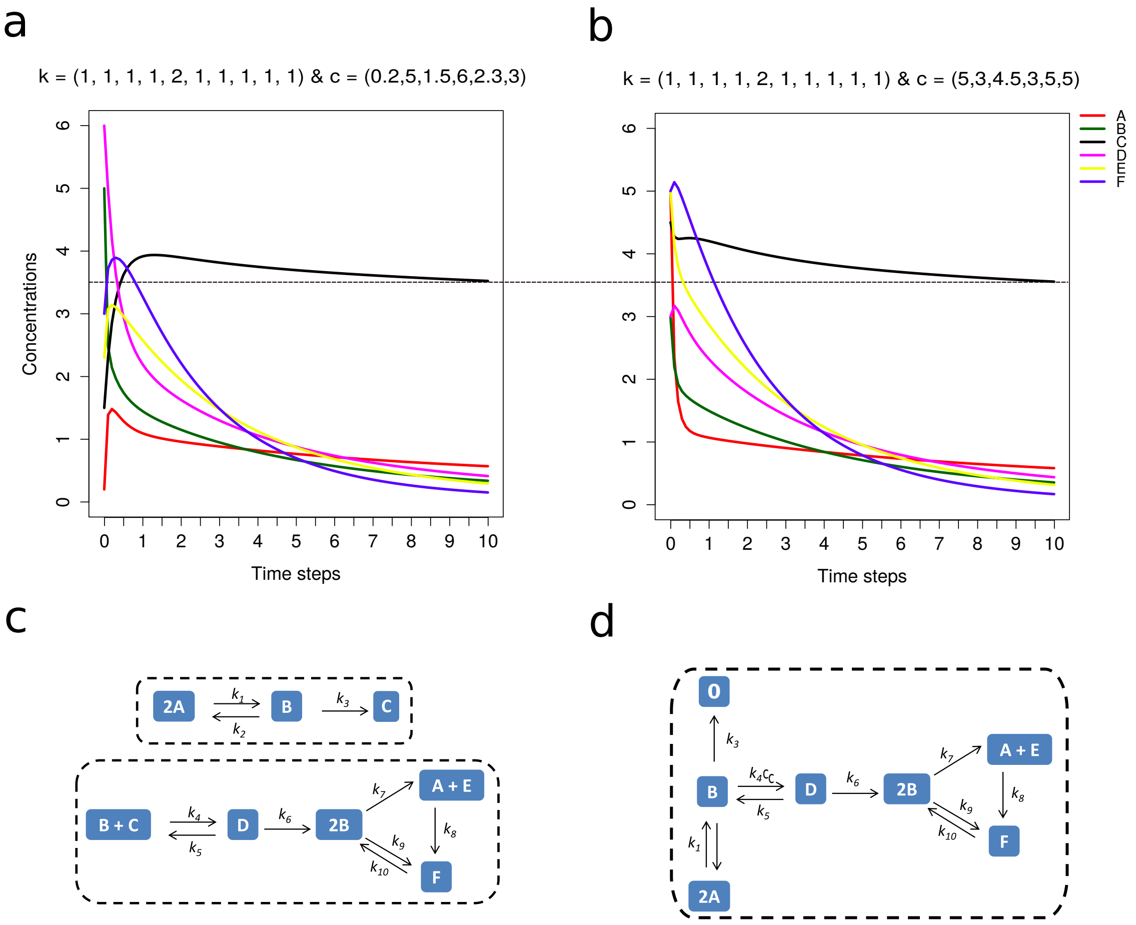

Supplementary Figure 4: **(a)** Concentration profiles of species (A to F) from network in panel c for the rate constants $k = (1, 1, 1, 1, 2, 1, 1, 1, 1, 1)$ and initial conditions $c = (0.2, 5, 1.5, 6, 2.3, 3)$. The network in panel c exhibits a positive steady state. **(b)** Concentration profiles of species (A to F) from the network in panel (c) for the rate constants $k = (1, 1, 1, 1, 2, 1, 1, 1, 1, 1)$ and initial conditions $c = (5, 3, 4.5, 3, 5, 5)$. In panels a and b species C tends to the same concentration profile of $c_C = 3$. **(c)** Network of biochemical reactions. Six species, denoted with capital letters (A to F), are interconverted through 10 irreversible reactions. **(d)** Network of biochemical reactions obtained upon removal of species C. The rate constants of the network in panel d are given by k_{-S} (see Supplementary Methods).

network in Fig. 4c has a positive steady state for $k_1 = 1, k_2 = 1, k_3 = 1, k_4 = 1, k_5 = 2, k_6 = 1, k_7 = 1, k_8 = 1, k_9 = 1, k_{10} = 1$. Since, the final concentration of C tends to a constant value as the time steps increase in both profiles, C has ACR. Moreover, the structural deficiency of the network in Fig. 4c is $\delta_s = 8 - 2 - 5 = 1$ and upon removal of species C from the network, we obtain the network in Fig. 4d, which is of structural deficiency $\delta_s^C = 7 - 1 - 5 = 1$. This is in line with the statement of Theorem 2.8. While, upon removal of species B we obtain the network given by Fig. 5, whose deficiency is $\delta_s^B = 6 - 1 - 5 = 0$. Therefore, species B is to

be ruled out from the set of possible ACR species. However, there exist some species, such as: A, D, E, and F, whose removal does not alter the deficiency of the original network but are not ACR (see analytical solution below). Therefore, our theorem does not provide conclusive answer about ACR for such species.

It can be analytically proven that species C in the network given by Fig. 4c, that the species C indeed exhibits ACR.

The system of ordinary differential equations describing the rate of change of the species concentrations in the network Fig. 4c is given by the following

$$\begin{aligned}
\frac{dc_A}{dt} &= -2k_1c_A^2 + 2k_2c_B + k_7c_B^2 - k_8c_Ac_E, \\
\frac{dc_B}{dt} &= k_1c_A^2 - (k_2 + k_3)c_B - k_4c_Bc_C + (k_5 + 2k_6)c_D - 2(k_7 + k_9)c_B^2 + 2k_{10}c_F, \\
\frac{dc_C}{dt} &= k_3c_B - k_4c_Bc_C + k_5c_D, \\
\frac{dc_D}{dt} &= k_4c_Bc_C - (k_5 + k_6)c_D, \\
\frac{dc_E}{dt} &= k_7c_B^2 - k_8c_Ac_E, \\
\frac{c_F}{dt} &= k_8c_Ac_E + k_9c_B^2 - k_{10}c_F.
\end{aligned} \tag{40}$$

By solving the system of ordinary equations given by Eq. (40), we have the following

$$\begin{aligned}
c_A^2 &= \frac{k_2}{k_1}c_B, \\
c_C &= \frac{(k_5 + k_6)k_3}{k_4k_6}, \\
c_D &= \frac{k_3}{k_6}c_B, \\
c_E^2 &= \frac{k_7^2k_1}{k_8^2k_2}c_B^3, \\
c_F &= \frac{k_9 + k_7}{k_{10}}c_B^2.
\end{aligned} \tag{41}$$

From the set of solutions provided by Eq. (41), we have that all the species concentrations depend on that of species B, except species C. This implies that the system shows ACR in species C. Let us consider the network in Fig. 6.

It can be seen in Fig. 6a and b that the system in Fig. 6c has a positive steady state for $k_1 = 1, k_2 = 5, k_3 = 3, k_4 = 3, k_5 = 3, k_6 = 2$. Since, the final concentration of E is identical in both profiles, E has ACR. Moreover, the structural deficiency of the network in Fig. 6c is $\delta_s = 6 - 2 - 3 = 1$ and upon removal of species E from the network, we obtain the network in Fig. 6d, which is of structural deficiency $\delta_s^E = 5 - 1 - 3 = 1$. This is in line with the statement of Theorem 2.8. Let us consider the network of deficiency 2 in Fig. 7c. For this

network, G is known to have the ACR property. The structural deficiency is $\delta_s = 13-4-7 = 2$ and upon removal of species G, the obtained network (see Fig. 7d), is of structural deficiency

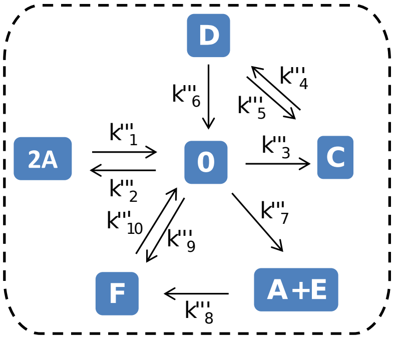

Supplementary Figure 5: **Network of biochemical reactions obtained upon removal of species B from Fig. 4c**. Five species A, C, D, E, F; six complexes and one linkage class.

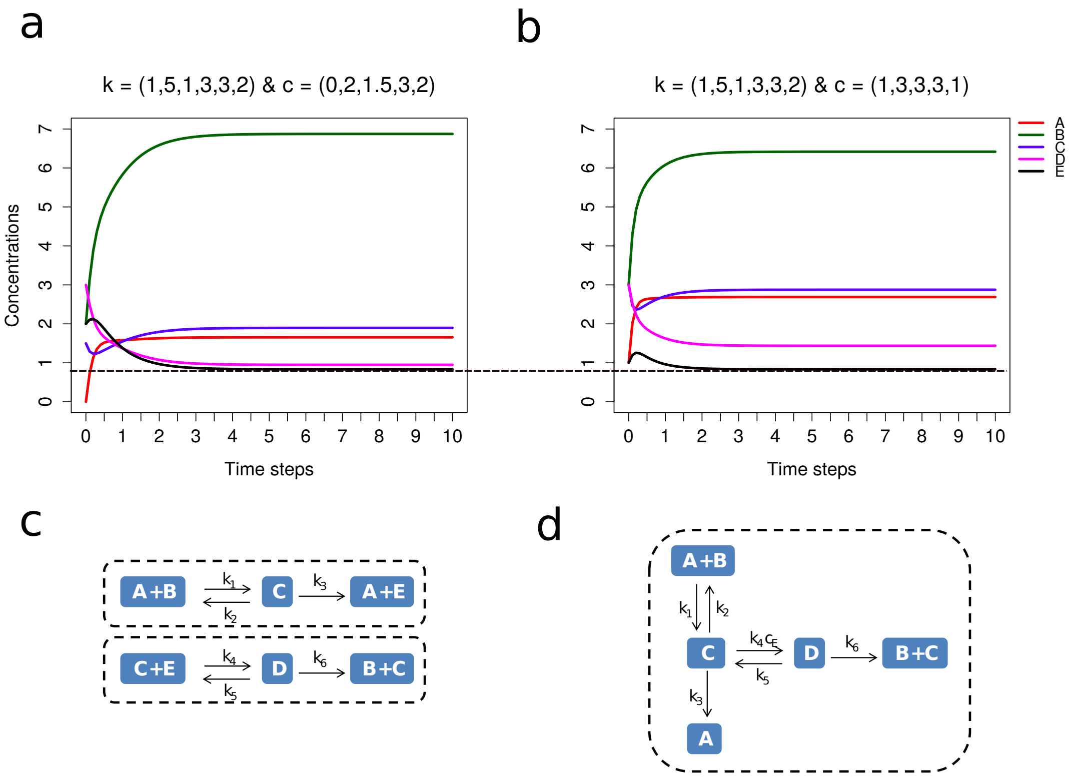

Supplementary Figure 6: **(a)** Concentration profiles of species (A to E) from network in panel c for the rate constants $k = (1, 5, 1, 3, 3, 2)$ and initial conditions $c = (0, 2, 1.5, 3, 2)$. The network in panel c exhibits a positive steady state. **(b)** Concentration profiles of species (A to E) from the network in panel (c) for the rate constants $k = (1, 5, 1, 3, 3, 2)$ and initial conditions $c = (1, 3, 3, 3, 1)$. In panels a and b species E has the same concentration profile. **(c)** Network of biochemical reactions. Five species, denoted with capital letters (A to E), are inter-converted through 6 irreversible reactions. **(d)** Network of biochemical reactions obtained upon removal of species E. The rate constants of the network in panel d are given by k_{-S} (see Supplementary Methods.

$\delta_s^G = 10 - 1 - 7 = 2$, which is in agreement with the theorem in the main text.

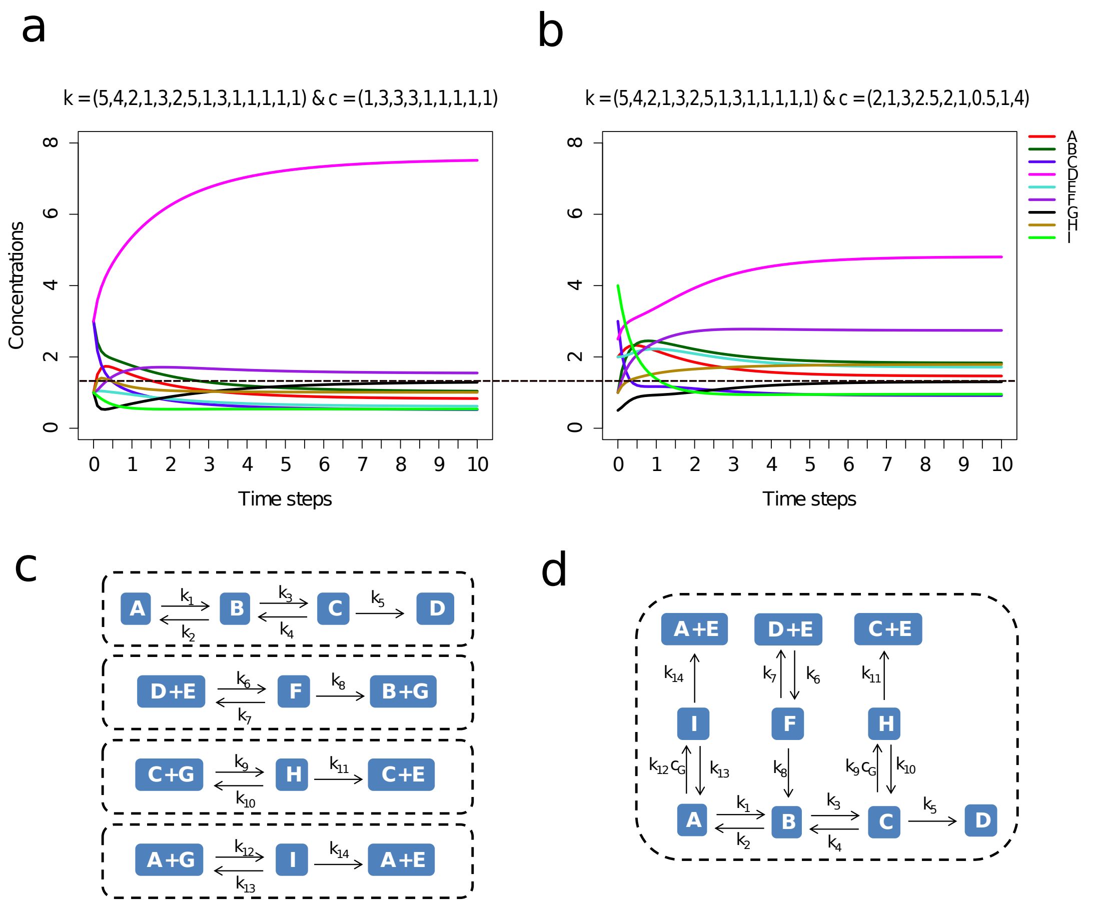

Supplementary Figure 7: **(a)** Concentration profiles of species (A to I) from network in panel c for the rate constants $k = (5, 4, 2, 1, 3, 2, 5, 1, 3, 1, 1, 1, 1, 1)$ and initial conditions $c = (1, 3, 3, 3, 1, 1, 1, 1, 1)$. The network in panel (c) exhibits a positive steady state. **(b)** Concentration profiles of species (A to I) from the network in panel c for the rate constants $k = (5, 4, 2, 1, 3, 2, 5, 1, 3, 1, 1, 1, 1, 1)$ and initial conditions $c = (2, 1, 3, 2.5, 2, 1, 0.5, 1, 4)$. In panels a and b species G has the same final concentration. **(c)** Network of biochemical reactions. Nine species, denoted with capital letters (A to I), are inter-converted through 14 irreversible reactions. **(d)** Network of biochemical reactions obtained upon removal of species G. The rate constants of the network in panel d are given by k_{-S} (see Supplementary Methods.

Supplementary references

1. R. P. Agaev and P. Yu. Chebotarev. The matrix of maximum out forests of a digraph and its applications. *Automation and remote control*, 2000.
2. Mikael Rørdam Andersen, Michael Lynge Nielsen, and Jens Nielsen. Metabolic model integration of the bibliome, genome, metabolome and reactome of aspecgillus niger. *Molecular systems biology*, 2008.
3. Anne Arnold and Zoran Nikoloski. Bottom-up metabolic reconstruction of arabidopsis and its application to determining the metabolic costs of enzyme production. *Plant Physiology*, 2014.
4. Natalie C. Duarte, Scott A. Becker, Neema Jamshidi, Ines Thiele, Monica L. Mo, Thuy S. Vo, Rohith Srivas, and Bernhard Ø Palsson. Global reconstruction of the human metabolic network based on genomic and bibliomic data. *Proceedings of the National Academy of Sciences*, 2007.
5. Martin Feinberg. Lectures on chemical reaction networks. *Notes of lectures given at the Mathematics Research Center, University of Wisconsin*, 1979.
6. Adam M Feist, Johannes CM Scholten, Bernhard Ø Palsson, Fred J Brockman, and Trey Ideker. Modeling methanogenesis with a genome-scale metabolic reconstruction of methanosarcina barkeri. *Molecular Systems Biology*, 2006.
7. Jeremy Gunawardena. Chemical reaction network theory for in-silico biologists. *June*, 2003.
8. Neema Jamshidi and Bernhard Ø Palsson. Investigating the metabolic capabilities of mycobacterium tuberculosis h37rv using the in silico strain inj 661 and proposing alternative drug targets. *BMC Systems Biology*, 2007.
9. Ali Khodayari, Ali R. Zomorrodi, James C. Liao, and Costas D. Maranas. A kinetic model of escherichia coli core metabolism satisfying multiple sets of mutant flux data. *Metabolic Engineering*, 2014.
10. Vinay Satish Kumara, James G. Ferry, and Costas D. Maranas. Metabolic reconstruction of the archaeon methanogen methanosarcina acetivorans. *BMC Systems Biology*, 2011.
11. Ani Manichaikul, Lila Ghamsari, Erik F. Y. Hom, Chenwei Lin, Ryan R. Murray, Roger L. Chang, S. Balaji, Tong Hao, Yun Shen, Arvind K. Chavali, Ines Thiele, Xin ping Yang Changyu Fan, Elizabeth Mello, David E. Hill, Marc Vidal, Kourosh Salehi-Ashtiani, and Jason A. Papin. Metabolic network analysis integrated with transcript verification for sequenced genomes. *Nature Methods*, 2009.
12. Juan Nogales, Bernhard Ø Palsson, and Ines Thiele. A genome-scale metabolic reconstruction of pseudomonas putida kt2440: ijn746 as cell factory. *BMC Systems Biology*, 2008.
13. Jeffrey D. Orth, Tom M. Conrad, Jessica Na, Joshua A Lerman, Hojung Nam, Adam M. Feist, and Bernhard Ø Palsson. A comprehensive genome-scale reconstruction of escherichia coli. *Molecular systems biology*, 2011.
14. Jeffrey D. Orth, Ines Thiele, and Bernhard Ø Palsson. What is flux balance analysis? *Nature Biotechnology*, 2010.

15. Lake-EE Quek and Lars K. Nielsen. On the reconstruction of the mus musculus genome-scale metabolic network model. *Genome Informatics*, 2008.

16. Robert Schuetz, Lars Kuepfer, and Uwe Sauer. Systematic evaluation of objective functions for predicting intracellular fluxes in escherichia coli. *Molecular Systems Biology*, 2007.

17. Guy Shinar, Ron Milo, Maria Rodriguez Martinez, and Uri Alon. Input–output robustness in simple bacterial signaling systems. *Proceedings of national academy of sciences of the USA*, 2007.

18. Judith A H Wodke, Jacek Puchalka, Maria Lluch-Senar, Josep Marcos, Eva Yus, Miguel Godinho, Ricardo Gutierrez-Gallego, Vitor A P Martins dos Santos, Luis Serrano, Edda Klipp, and Tobias Maier. Dissecting the energy metabolism in mycoplasma pneumoniae through genome-scale metabolic modeling. *Molecular systems biology*, 2013.

19. Ying Zhang, Ines Thiele, Dana Weekes, Zhanwen Li, Lukasz Jaroszewski, Krzysztof Ginalski, Ashley M. Deacon, John Wooley, Scott A. Lesley, Ian A. Wilson, Bernhard Ø Palsson, Andrei Osterman, and Adam Godzik. Three-dimensional structural view of the central metabolic network of thermotoga maritima. *Science*, 2009.
